# CXCL10/CXCR3 axis is associated with disease activity and the development of mucocutaneous lesions in patients with Behçet’s disease

**DOI:** 10.1038/s41598-017-15189-9

**Published:** 2017-11-07

**Authors:** Sang Jin Lee, Shin Eui Kang, Eun Ha Kang, Byoong Yong Choi, Katherine Masek-Hammerman, Jameel Syed, Yutian Zhan, Kathleen Neff-Phillips, Jin Kyun Park, Eun Young Lee, Eun Bong Lee, Yeong Wook Song

**Affiliations:** 1 Division of Rheumatology, Department of Internal Medicine, Seoul National University Hospital, Seoul, Republic of Korea; 2 Department of Molecular Medicine and Biopharmaceutical Sciences, Graduate School of Convergence Science and Technology, and College of Medicine, Medical Research Center, Seoul National University, Seoul, Republic of Korea; 3 Division of Rheumatology, Department of Internal Medicine, Seoul National University Bundang Hospital, Seoul, Republic of Korea; 4 Division of Rheumatology, Department of Internal Medicine, Seoul Medical Center, Seoul, Republic of Korea; 5 Drug Safety Research and Development, Pfizer Inc, Andover, MA. USA; 6 Inflammation and Immunology Research Unit, Pfizer Inc, Cambridge, MA. USA

## Abstract

The objective of this study was to investigate CXC chemokines and its receptor in patients with Behcet’s disease (BD) and their associations with disease activity. Blood samples were collected from 109 BD patients and 36 age- and sex-matched healthy controls (HCs). Twenty-two follow-up blood samples were collected in BD patients. Serum CXC chemokines (CXCL1, CXCL8, CXCL9, CXCL10, CXCL12, CXCL13 and CXCL16) and cell surface marker expression (CD3, CD4 and CXCR3) in peripheral blood mononuclear cells (PBMCs) were assayed. Clinical features including disease activity were evaluated at the time of blood collection. CXCR3 expression in skin and intestinal lesions from BD patients and HCs was assessed via immunohistochemistry. Serum CXCL10 levels were correlated with disease activity in terms of Behçet’s Disease Current Activity Form (BDCAF) (p < 0.001). In follow-up BD patients, changes in serum CXCL10 levels tended to be correlated with those of BDCAF. The percentage of CXCR3 expression in CD3-positive cells in PBMCs was inversely correlated with serum CXCL10 levels in BD patients (p = 0.022). By immunohistochemistry, the number of CXCR3-positive mononuclear cells was higher in skin and intestinal lesions of BD patients than in those of HCs. These results suggest that the CXCL10/CXCR3 axis may contribute to the pathogenesis of BD.

## Introduction

Behçet’s disease (BD) is a chronic, multisystem inflammatory syndrome characterized by recurrent oral ulcers, genital ulcers, cutaneous lesions and uveitis^1^. In addition, blood vessels, the nervous system, joints and the gastrointestinal tract may be involved less frequently^2^. Although the etiology of BD is unclear, the findings of a genome-wide association study suggest that both innate and adaptive immunity are dysregulated in BD^3,4^. The innate sensing and processing of danger signals and subsequent polarization toward the T helper (Th)1/Th17 pathway may play critical roles in driving pathogenesis^3–5^.

Chemokines are relatively small, secreted proteins produced by various cells that control leukocyte recruitment to inflammatory sites. Based on patterns of cysteine residues, chemokines were categorized into four subfamilies (CC, CXC, XC and CX3C). Of those, CC and CXC chemokines form two large gene clusters^6^. Chemokines and their receptors participate in homeostasis of the immune system^7^. The CXCL10/CXCR3 and CXCL13/CXCR5 axes are associated with disease activity in several autoimmune diseases including rheumatoid arthritis (RA), systemic lupus erythematosus (SLE) and adult onset Still’s disease^8–12^. Moreover the CXCL12/CXCR4 axis contributes to development of RA and SLE^13,14^.

It was reported that CXCL8 levels in serum, CXCL10 levels in aqueous humor and cerebrospinal fluid (CSF) were significantly increased in BD patients compared to controls^5,15–17^. CXCR2, which is the receptor for CXCL8, was a prominent chemokine receptor on neutrophils and CXCR3 was highly expressed on memory T cells and/or activated Th1 cells^18,19^. But it remains to be investigated whether chemokines and their concomitant receptors are important in the pathogenesis and disease activity of BD. The objective of this study was to investigate CXC chemokines and its receptor in patients with BD and their associations with disease activity. Multiple serum chemokines were assayed at baseline and follow-up in patients with BD and their association with disease activity were evaluated. Subsequently, we analyzed cell surface marker expression in peripheral blood mononuclear cells (PBMCs) and immunohistochemistry (IHC) of tissue lesions in patients with BD.

## Results

### Baseline clinical characteristics of patients with BD and healthy controls (HCs)

The mean age of BD patients in this study was 46.6 ± 12.5 years, and 63.3% were female. The disease duration after BD diagnosis was 7.8 ± 7.1 years. The principal clinical symptoms of BD patients were oral ulcer (OU) (72.5%), genital ulcer (GU) (17.4%), erythema nodosum (EN) (37.6%) and current uveitis (6.4%) over the previous 4 weeks. Scores of Behcet’s Disease Current Activity Form (BDCAF) (0–12) and Behcet’s Syndrome Activity Score (BSAS) (0–100) were 2.8 ± 1.5 and 24.9 ± 14.9, respectively. Sixty-nine percent of patients were on colchicine, 40% on corticosteroids (daily dose of prednisolone or its equivalent, 3.3 ± 6.6 mg) and none of the patients had prior exposure to biologics (Table 1).

**Table 1:** Clinical and laboratory parameters of Behçet’s disease patients and healthy controls.

	BD patients (n = 109)	Healthy controls (n = 36)
Age (years)	46.6 ± 12.5	46.8 ± 12.4
Sex, female (%)	69 (63.3)	22 (61.1)
Disease duration, years	7.8 ± 7.1	N/A
ESR (mm/h)	23.1 ± 16.6	N/A
CRP (mg/ml)	0.4 ± 0.8	N/A
Clinical manifestations		
Oral ulcer	79 (72.5)	N/A
Genital ulcer	19 (17.4)	N/A
Erythema nodosum	41 (37.6)	N/A
Folliculitis	43 (40.2)	N/A
Uuveitis, n (%)	7 (6.4)	N/A
Arthralgia, n (%)	48 (44.0)	N/A
Colitis, n (%)	6 (5.5)	N/A
BDCAF (0–12)	2.8 ± 1.5	N/A
BSAS (0–100)	24.9 ± 14.9	N/A
Treatment modality		
Colchine, n (%)	75 (68.8)	N/A
Corticosteroid, n (%)	44 (40.4)	N/A
Daily dose of prednisolone or its equivalent (mg)	3.3 ± 6.6	N/A
Immunosuppressant, n (%)	43 (39.4)	N/A

Data are expressed as means ± SD for continuous variables or percentages for categorical variables. Current colitis was defined by diagnosis by colonoscopy 6 months prior to blood samples. BD, Behçet’s disease; ESR, erythrocyte sedimentation rate; CRP, C-reactive protein; BDCAF, Behçet’s Disease Current Activity Form; BSAS, Behçet’s Syndrome Activity Score; Immunosuppressant, sulfasalazine or cyclosporine or tacrolimus or azathioprine.

### Baseline serum chemokine levels in patients with BD and HCs

**Figure 1:**
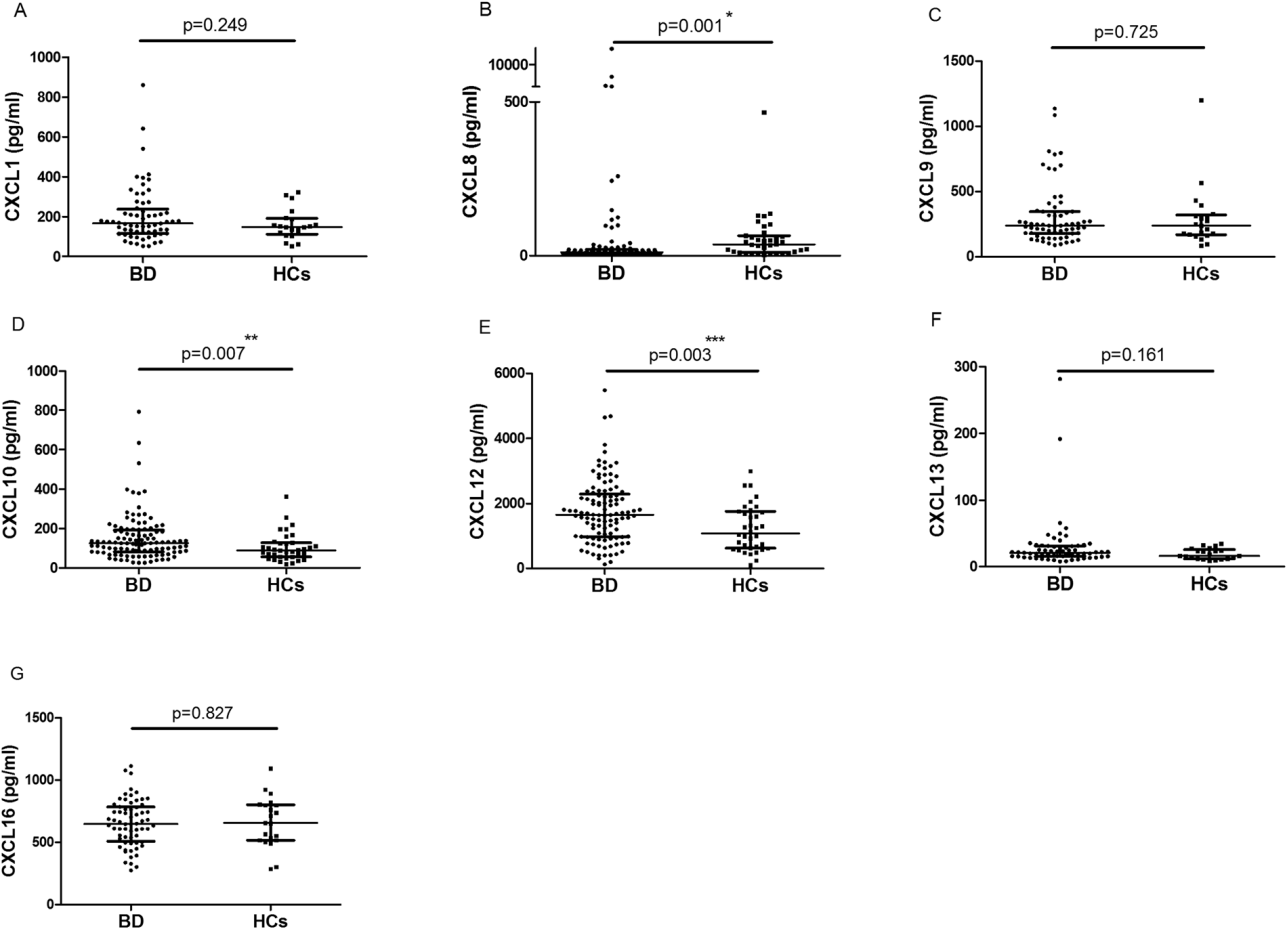
Serum chemokine levels at baseline in BD patients and HCs. Serum levels of CXCL1 (**A**), CXCL8 (**B**), CXCL9 (**C**), CXCL10 (**D**), CXCL12 (**E**), CXCL13 (**F**) and CXCL16 (**G**) in BD and HCs are shown. Each dot represents an individual value and bars represent the median values and interquartile ranges. P-value was assessed by Mann-Whitney U test. BD, Behçet’s disease; HCs, healthy controls; *corrected p = 0.007; **corrected p = 0.049; ***corrected p = 0.021 by Bonferroni’s correction.

Serum levels of CXCL8 (mean ± SD 230 ± 1656, median 11 pg/ml vs. 57 ± 80, median 36 pg/ml, respectively; p = 0.001) CXCL10 (153 ± 118, median 127 pg/ml vs. 105 ± 72, median 90 pg/ml, respectively; p = 0.007) and CXCL12 (1771 ± 987, median 1656 pg/ml vs. 1234 ± 713, median 1086 pg/ml, respectively; p = 0.003) were significantly higher in blood collected at the time of initial evaluation from BD patients than HCs. However serum levels of CXCL1 (205 ± 143, median 167 pg/ml vs. 160 ± 76, median 147 pg/ml, respectively; p = 0.249), CXCL9 (313 ± 232, median 239 pg/ml vs. 294 ± 239, median 238 pg/ml, respectively; p = 0.725), CXCL13 (30 ± 40, median 21 pg/ml vs. 20 ± 8, median 16 pg/ml, respectively; p = 0.161) and CXCL16 (656 ± 187, median 648 pg/ml vs. 663 ± 202, median 657 pg/ml, respectively; p = 0.827) were not different between BD patients and HCs (Fig. 1).

### Serum CXCL10 levels and clinical manifestations in patients with BD

CXCL10 levels from blood collected at the time of initial evaluation were significantly higher in patients with OU (mean ± SD 162 ± 106, median 136 pg/ml vs. 130 ± 145, median 99 pg/ml, respectively; p = 0.008), GU (181 ± 90, median 151 pg/ml vs. 147 ± 123, median 124 pg/ml, respectively; p = 0.022) or EN (193 ± 144, median 151 pg/ml vs. 129 ± 92, median 104 pg/ml, respectively; p = 0.001) than in patients without. However, CXCL10 levels were not significantly different in patients with enteritis, folliculitis or uveitis compared to patients without (p = 0.651, p = 0.557 and p = 0.066, respectively) (Supplementary Fig. 2).

### Correlation between serum chemokine levels and disease activity in patients with BD

**Figure 2:**
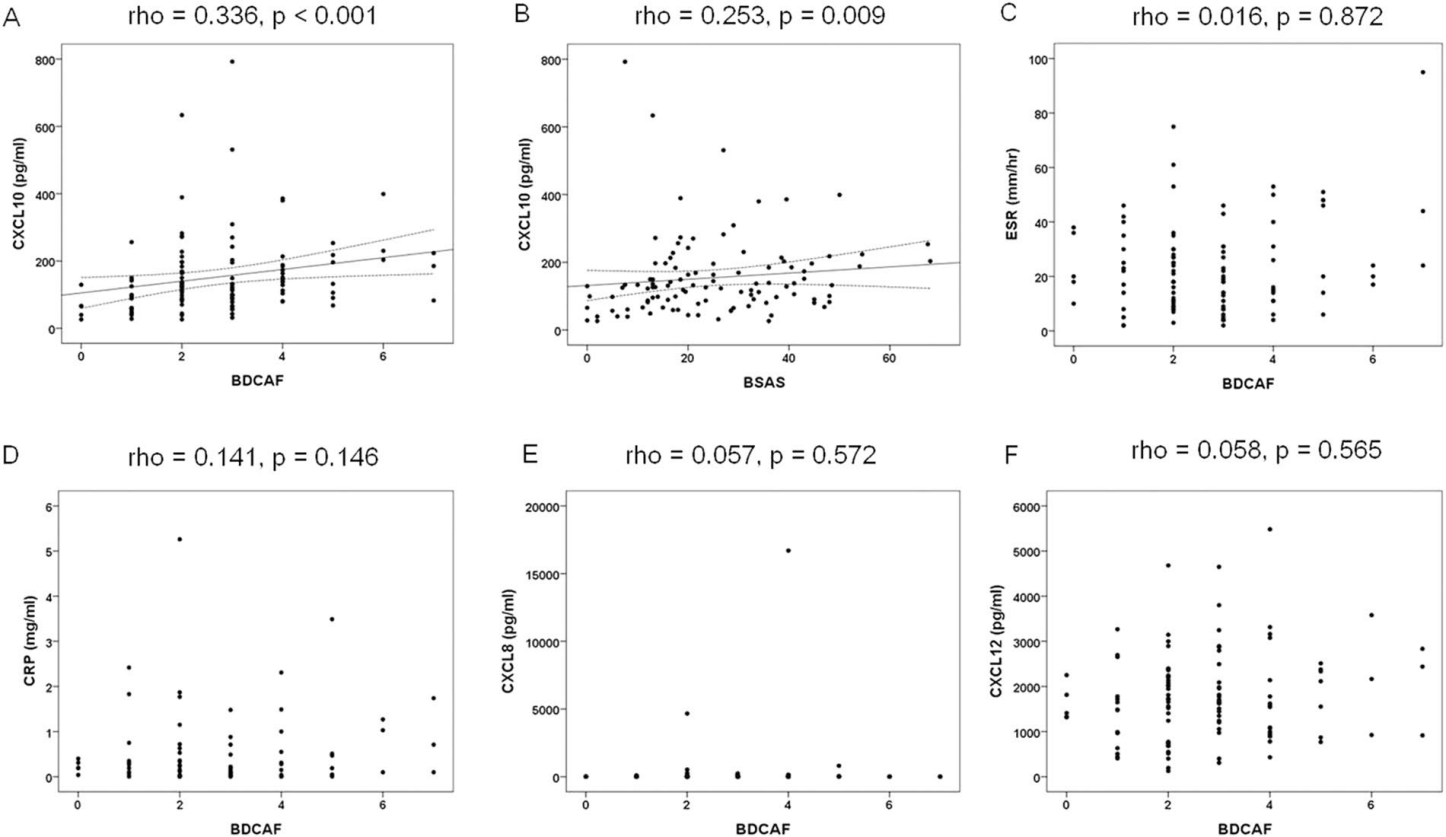
Correlations between serum chemokine levels and disease activity at baseline in patients with BD. Serum CXCL10 levels were significantly correlated with disease activity in terms of both the BDCAF (**A**) (n = 108) and BSAS (**B**) (n = 106). However, ESR (**C**) (n = 100), CRP (**D**) (n = 100), CXCL8 (**E**) (n = 108) and CXCL12 (**F**) (n = 108) levels were not significantly correlated with disease activity in terms of the BDCAF. Each dot represents an individual value and dotted lines represent the 95% confidence interval ranges. Correlation coefficient and p-value were analyzed by Spearman’s correlation test. BD, Behçet’s disease; ESR, erythrocyte sedimentation rate; CRP, C-reactive protein; BDCAF, Behçet’s Disease Current Activity Form; BSAS, Behçet’s Syndrome Activity Score.

Baseline serum CXCL10 levels were significantly correlated with disease activity in terms of both BDCAF and BSAS (rho = 0.336, p < 0.001 and rho = 0.253, p = 0.009, respectively) at the time of initial evaluation (Fig. 2A,B). However, erythrocyte sedimentation rate, C-reactive protein, CXCL8 and CXCL12 levels were not significantly correlated with disease activity in terms of both BDCAF and BSAS (Fig. 2C–F) (BSAS data not shown). Follow-up samples from 22 BD patients were analyzed to evaluate changes in serum CXCL10 levels and disease activity markers compared to baseline. Changes in serum CXCL10 levels during disease course tended to be correlated with those of BDCAF (rho = 0.425, p = 0.048: corrected p = 0.096) (Table 2).

**Table 2:** Correlation between changes in serum CXCL10 levels and those of disease activity markers in follow-up BD patients (n = 22) compared to baseline.

Disease activity marker	Correlation coefficient (delta CXCL10)	
	rho	P- value
Delta BDCAF	0.425	0.048*
Delta BSAS	0.094	0.684
Delta CXCL8	−0.059	0.793
Delta CXCL12	0.342	0.119
Delta ESR	−0.049	0.828
Delta CRP	0.086	0.705

Follow-up samples from 22 BD patients (after 9.8 ± 6.7 months, mean ± SD) were analyzed to evaluate changes in serum CXCL10 levels and disease activity markers compared to baseline. Correlation coefficient and p-value were analyzed by Pearson’s correlation test. BD, Behçet’s disease; BDCAF, Behçet’s Disease Current Activity Form; BSAS, Behçet’s Syndrome Activity Score; ESR, erythrocyte sedimentation rate; CRP, C-reactive protein; *corrected p = 0.096 by Bonferroni’s correction.

### CXCR3 expression on CD3-T cells and correlation with serum CXCL10 levels

**Figure 3:**
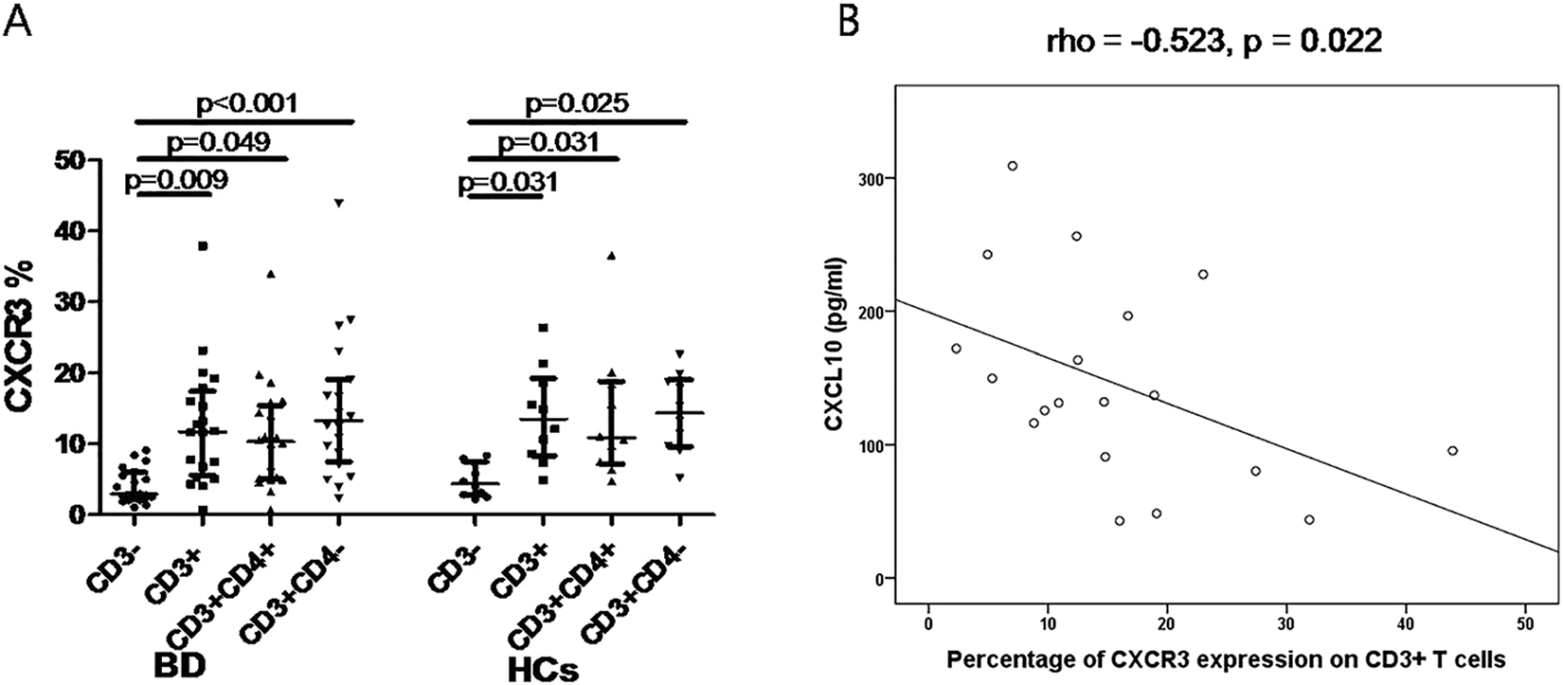
CXCR3 expression in lymphocytes from PBMCs and correlation of CXCR3-positive cells on CD3 T cells with serum CXCL10 levels. CXCR3 expression was significantly increased in CD3-positive cells compared to CD3-negative cells in lymphocytes of both BD patients and HCs (**A**). Percentages of CXCR3 expression in CD3-positive cells were inversely correlated with serum CXCL10 levels in patients with BD (**B**). Each dot represents an individual value and bars represent the median values and interquartile ranges. P-value by Mann-Whitney U test. Correlation coefficient and p-value were analyzed by Spearman’s correlation test. PBMCs, peripheral blood mononuclear cells; BD, Behçet’s disease; HCs, healthy controls.

CXCR3 expression was significantly higher on CD3-positive vs. CD3-negative cells in lymphocytes of PBMCs in both BD patients (p = 0.009) and HCs (p = 0.031). However, there was no difference in CXCR3 expression on CD3-positive cells or CD3-positive CD4-positive cells or CD3-positive CD4-negative cells between BD patients and HCs (p = 0.481, p = 0.356 and p = 0.792, respectively) (Fig. 3A). The percentage of CXCR3 expression on CD3-positive cells was inversely correlated with serum CXCL10 levels in BD patients (rho = −0.523, p = 0.022) (Fig. 3B). However this inverse correlation was not seen in HCs (data not shown).

### CXCR3 expression in the skin and intestine of BD patients and HCs

**Figure 4:**
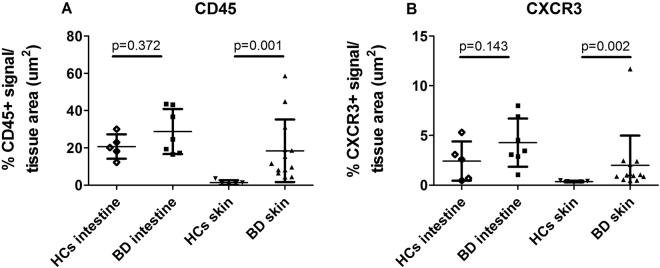
CD45 and CXCR3 expression in the skin and intestine of patients with BD and HCs. The percentages of CD45-positive cells (**A**) and CXCR3-positive cells (**B**) were significantly higher in skin samples of BD patients (n = 13) than in those of HCs (n = 5). P-value by Mann-Whitney U test. BD, Behçet’s disease; HCs, healthy controls.

By IHC, the percent area positive for CD45 (a pan-leukocyte marker) and CXCR3 expression was significantly higher in skin samples from BD patients than in those from HCs (p = 0.001 and p = 0.002, respectively). Although not statistically significant, the areas of reactivity for these markers tended to be higher in intestine samples from some BD patients compared to HCs (Fig. 4). The skin and intestine of BD patients were infiltrated by mostly mononuclear cells and neutrophils. Neutrophils, which are CXCR3-negative, were included in the CD45 counts (Supplementary Fig. 3). The infiltrating CXCR3-positive cells in both intestine and skin were characterized by predominantly mononuclear appearance, and were most consistent with lymphocytes in morphology.

## Discussion

To investigate the role of CXC chemokines and its receptor in BD patients, we measured levels of CXC chemokines (CXCL1, CXCL8, CXCL9, CXCL10, CXCL12, CXCL13 and CXCL16) and compared them with disease activity and clinical manifestations. Subsequently, we analyzed cell surface marker expression in PBMCs and IHC of tissue lesions.

To our knowledge, this is the first report of a significant correlation between serum CXCL10 levels and disease activity as well as mucocutaneous lesions in BD patients. In agreement with published data, the majority of CXCR3-positive cells in the peripheral blood of BD patients and HCs resided within the CD3-positive T cell population^20,21^. No significant difference in CXCR3 frequency was observed on T cells from BD patients compared to HCs. Although no increase in CXCR3 frequency was seen in the peripheral blood, a significant increase in the percentage of CXCR3-positive cells was observed in skin lesions from BD patients compared to HCs. These data, along with the inverse relationship between serum CXCL10 levels and the percentage of CXCR3-positive T lymphocytes in the blood of BD patients, suggests a role for the CXCL10/CXCR3 axis in the trafficking of cells to the site of inflammation.

Chemokine levels in BD and their association with disease have been explored by others previously. Levels of CXCL10 in aqueous humor were significantly higher from BD-associated uveitis than healthy controls, and correlated with disease activity of uveitis^15^. In the CSF, CXCL10 levels were also significantly higher in neuro-BD than non-inflammatory neurological diseases, and correlated with the leukocyte count^16,17^. Moreover, significantly increased levels of CXCL10 were noted in skin pathergy reactions of BD patients compared to normal skin sites^22^. Conversely it has been reported that serum CXCL10 levels were not significantly different between patients with BD and healthy controls, however the majority of the BD patients in this study were on biologic treatment at baseline^5^ which may impact the chemokine levels.

A gradient of CXCL10 levels is thought to be required for optimal differentiation of naïve T cells into Th1 cells in lymph nodes^23^. For example, serum CXCL10 levels are correlated with high expression of CXCR3 in pancreatic lymph nodes of type 1 diabetes patients suggesting a role of the CXCL10/CXCR3 axis in the accumulation of Th1 cells into the lymph nodes^24^. T lymphocytes utilize a localized gradient of CXCR3 ligands (e.g., CXCL9 and CXCL10), which are produced by infected monocytes, for entrance into virus-infected or inflamed tissues^25^. Consistent with our data, CXCR3-expressing T lymphocytes as well as levels of Th1 cytokines including interleukin (IL)−8, IL-12 and interferon γ have been shown to be increased in oral ulcers of BD patients compared to healthy controls^26,27^. These observations support the hypothesis that a gradient of CXCL10 may play an important role in polarization toward the Th1 pathway and enable CXCR3-expressing T lymphocytes to migrate into targeted tissues.

Our study had several limitations. First, many patients were on low dose corticosteroids. However, there were no statistical differences in chemokine levels and disease activity between BD patients who were taking corticosteroids and those who were not taking them (data not shown). Second, although no statistically significant difference was seen in the intestine of BD patients compared to HCs, a trend toward greater CD45 and CXCR3 expression was observed in some individual BD samples. This lack of significance may have been due to the low sample number and relatively high resident leukocyte population present in the lamina propria and gut-associated lymphoid tissue in the intestine. More samples may be needed to determine the significance of this trend. Third, we could not examine oral ulcer and genital ulcer biopsies from BD patients for CXCR3 expression. However, it was reported that numbers of CXCR3 expressing T lymphocytes were increased in oral ulcers from BD patients compared to healthy controls^26^. Fourth, there was no difference in CXCR3 expression on CD3-positive cells or CD4-positive cells between BD patients and HCs in our study. Although it is known that CXCR3 is selectively and highly expressed on memory T cells or Th1 effector cells^19^, CXCR3 on these populations was not looked at in this study

In conclusion, our study suggests that the CXCL10/CXCR3 axis may contribute to the pathogenesis of BD, and particularly in the development of mucocutaneous lesions. The serum CXCL10 level may be used as a serological biomarker of disease activity in BD patients.

## Patients and Methods

### Patients

One hundred and nine patients with BD, who received medical care at Seoul National University Hospital, and 36 age- and sex-matched HCs were enrolled in this study. This was a prospective follow-up study for BD patients who were diagnosed according to the criteria of the International Study Group for Behçet’s Disease^28^. BD patients who were ever treated with a biologic and/or had active infection states at the time of evaluation were excluded from the analysis. Patients were evaluated at the time of blood collection regarding their disease activity using both BDCAF and BSAS, clinical manifestations and concurrent medications^29,30^. Disease activity and quantification of lesion (OU, GU, EN and pustule) by patients over the previous 4 weeks were recorded. Among these patients 22 were followed for evaluation of serum chemokines and disease activity after 9.8 ± 6.7 months (mean ± SD). Serum samples were stored at −80 °C until chemokine assay. Heparinized blood samples were used immediately to assess cell surface expression of chemokine receptor (Supplementary Fig. 1). Skin and intestine sections investigated by IHC were samples from an unrelated set of 20 BD patients and 10 HCs that were banked at Seoul National University Hospital. Informed consent was provided from all individual participants included in the study. The protocol was approved by the Institutional Review Board at Seoul National University Hospital. The study was conducted in full concordance with the principles of the declaration of Helsinki.

### Measurement of serum chemokines

Serum chemokines at baseline (n = 109) were assayed for neutrophil chemoattractants (CXCL1 and CXCL8) and lymphocyte chemoattractants (CXCL9, CXCL10, CXCL12, CXCL13 and CXCL16) using a multiplex assay (Bio-Rad Laboratories Inc., Hercules, CA, USA). Serum chemokines of CXCL8, CXCL10 and CXCL12 at the time of follow-up (n = 22) were measured using the same methods.

### Analysis of chemokine receptor expression in PBMCs

PBMCs were separated from heparinized blood by Ficoll-gradient methods. The cells (1×10^6^) were stained with the following antibodies: phycoerytherin (PE)-labeled anti-human CD3 (clone: UCHT1, 555333 BD Bioscience, San Jose, CA, USA), allophycocyanin (APC)-labeled anti-human CD4 (clone: RPA-T4, 555349 BD Bioscience, San Jose, CA, USA), peridinin chlorophyll-cyanine 5.5 (PerCP-Cy5.5)-labeled anti-human CXCR3 (clone: 1C6/CXCR3, 560832 BD Bioscience, San Jose, CA, USA). Cells were stained at 4 °C for 20 min, and fixed with 1% paraformaldehyde. Flow cytometry was performed using a BD LSRFortessa (Becton Dickinson, Franklin Lakes, NJ, USA) and results were evaluated using FlowJo software (TreeStar Inc., Ashland, OR, USA).

### Immunohistochemistry and quantification

IHC for CXCR3/CD183 (clone: 1C6, 557183 BD Biosciences, San Jose, CA) and CD45/LCA (clone: × 16/99, PA0042, Leica Microsystems, Buffalo Grove, IL) was performed on formalin-fixed paraffin-embedded skin and intestine sections from 20 BD patients (n = 7 intestine, n = 13 skin) and 10 HCs (n = 5 intestine, n = 5 skin). Tissue sections were banked samples at Seoul National University Hospital unrelated to the enrolled BD patients. IHC was performed on a Leica BOND RX automated IHC platform. CXCR3/CD183 IHC assays were detected using the Bond Refine Compact Polymer detection system (horse radish peroxidase anti-mouse DAB, DS9800; Leica Biosystems). CD45 was detected using Bond Polymer Refine Red Detection system (alkaline phosphatase red anti-mouse, DS9390). Slides were counter stained with hematoxylin and cover slipped. The area of positive signal in stained sections of each biopsy was quantified using Definiens Tissue Studio (Definiens AG, Munich, Germany) and normalized to the measured area of biopsy (excluding white space) on each slide.

### Statistical analysis

Baseline clinical data were presented as means ± SD. Levels of chemokines and CXCR3 expression were presented as median ± interquartiles. Differences among continuous variables were assessed using the Mann-Whitney test. The correlations between levels of CXCL10 and disease activity or frequency of symptoms or levels of chemokine receptor expression were evaluated using Spearman’s or Pearson’s correlation test. P values < 0.05 were considered to indicate statistical significance. In addition, for the baseline analysis of multiple chemokines and the correlational analysis with disease activity, Bonferroni’s correction was done. All statistical analyses were performed using IBM SPSS (statistics version 19.0, Chicago, IL, USA).

## Electronic supplementary material

Supplementary data
