# Supplementary material for: CXCL10/CXCR3 axis is associated with disease activity and the development of mucocutaneous lesions in patients with Behçet’s disease
Source: Sci Rep. 2017 Nov 7;7:14720. doi: 10.1038/s41598-017-15189-9 (PMC5677118; doi:10.1038/s41598-017-15189-9)
Supplement: Supplementary file 1 — Supplementary data [file 41598_2017_15189_MOESM1_ESM.pdf]

CXCL10/CXCR3 axis is associated with disease activity and the development of mucocutaneous lesions in patients with Behçet's disease

Sang Jin Lee[1,2], Shin Eui Kang[2], Eun Ha Kang[3], Byoong Yong Choi[4], Katherine Masek-Hammerman[5], Jameel Syed[5], Yutian Zhan[5], Kathleen Neff-Phillips[6], Jin Kyun Park[1,2], Eun Young Lee[1], Eun Bong Lee[1], Yeong Wook Song[1,2]

[1]Division of Rheumatology, Department of Internal Medicine, Seoul National University Hospital, Republic of Korea, [2]Department of Molecular Medicine and Biopharmaceutical Sciences, Graduate School of Convergence Science and Technology, and College of Medicine, Medical Research Center, Seoul National University, [3]Division of Rheumatology, Department of Internal Medicine, Seoul National University Bundang Hospital, Republic of Korea, [4]Division of Rheumatology, Department of Internal Medicine, Seoul Medical Center, Seoul, Republic of Korea, [5]Drug Safety Research and Development, Pfizer Inc, Andover, MA. USA. [6]Inflammation and Immunology Research Unit, Pfizer Inc, Cambridge, MA. USA.

Corresponding author:

Yeong Wook Song, MD

Dept of Internal Medicine, Seoul National University Hospital

Yungun-dong 28, Jongno-gu, Seoul, 110-744, KOREA

FAX:822-762-9662, Phone:822-2072-2234, E-mail:ysong@snu.ac.kr

Supplementary Fig. 1

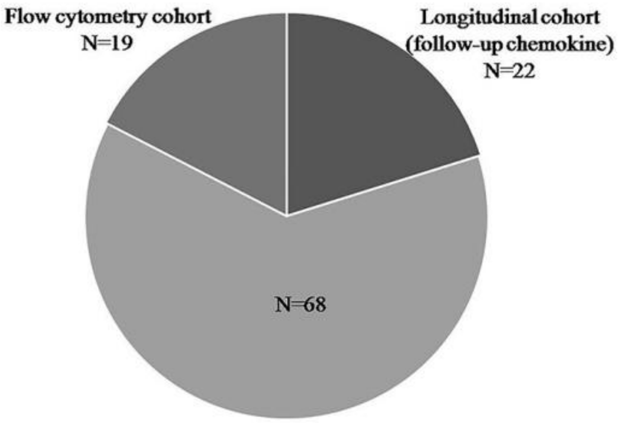

Total chemokine cohort, N=109

Supplementary Fig. 2

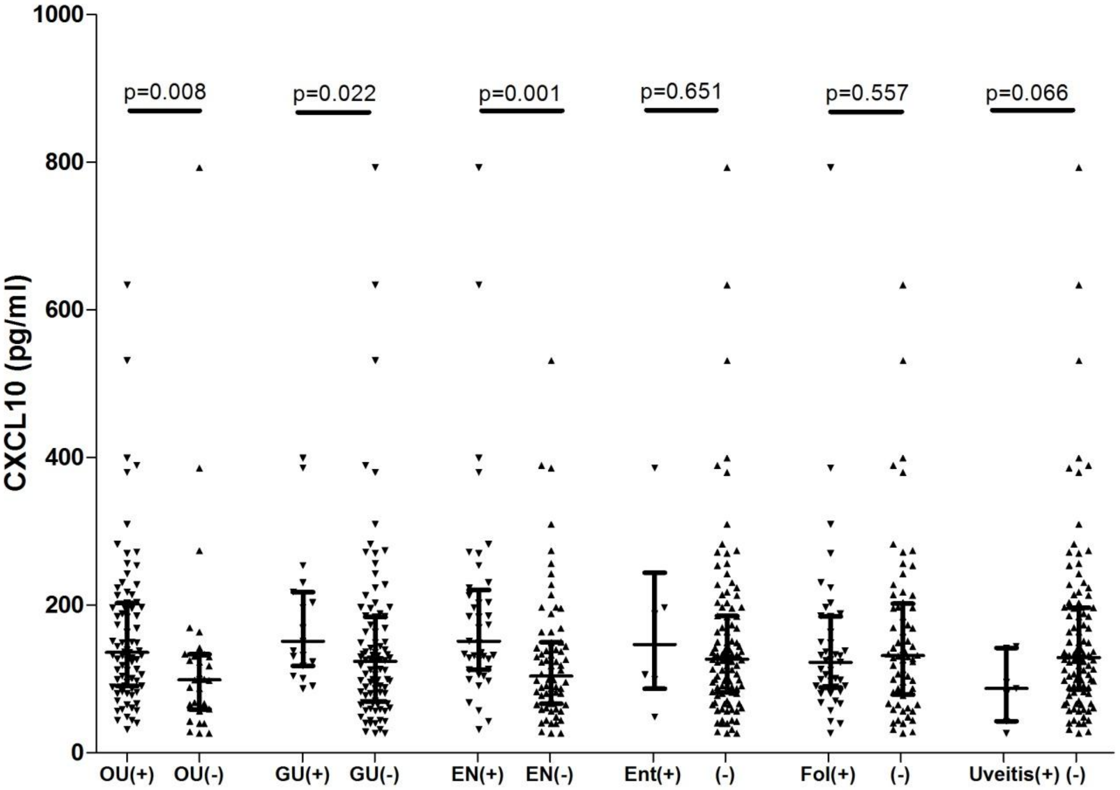

Supplementary Fig. 3

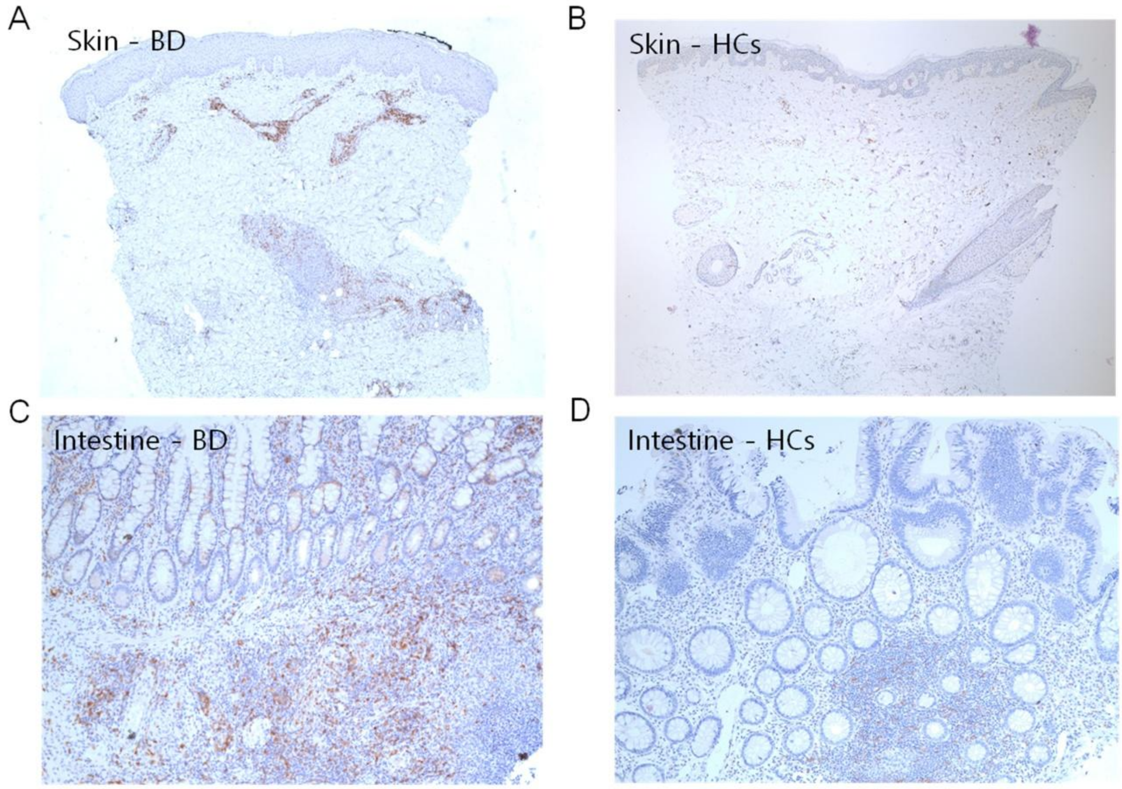

Supplementary figure legends

Supplementary Fig. 1 Distribution of enrolled patients with BD. Serum chemokines at baseline were assayed for 109 patients with BD. Among these patients 19 were assessed for cell surface expression of CXCR3 (flow cytometry cohort) and 22 were followed for evaluation of serum chemokines (longitudinal cohort). There were no statistical differences in disease activity between patients who were examined for baseline chemokine and patients who were examined for flow cytometry or for follow-up chemokine. BD, Behçet's disease.

Supplementary Fig. 2 Serum CXCL10 levels and clinical manifestations in patients with BD. Serum CXCL10 levels from blood collected at initial patient evaluation were significantly higher in patients with OU or GU or EN than in patients without. Each dot represents an individual value and bars represent the median values and interquartile ranges. P-value was assessed by Mann-Whitney U test. OU, oral ulcer; GU, genital ulcer; EN, erythema nodosum; Ent, enteritis; Fol, folliculitis.

Supplementary Fig. 3 CXCR3 expression in the skin and intestine of representative BD patients and HCs. CXCR3-positive cells were seen in skin (original magnification, 40x) (A, B) and intestine (original magnification, 100x) (C, D) of both BD patients (A, C) and HCs (B, D). CXCR3-positive cells in skin and intestine had predominantly mononuclear appearance. BD, Behçet's disease; HCs, healthy controls
